# Effects of vitamin D levels on lipid, glucose profiles and depression in perimenopausal women

**DOI:** 10.1192/j.eurpsy.2025.1365

**Published:** 2025-08-26

**Authors:** D. Schneider-Matyka, A. M. Cybulska, K. Rachubińska, D. Kostecka, M. Nowak, E. Grochans

**Affiliations:** 1 Department of Nursing; 2 Pomeranian Medical University in Szczecin, Szczecin, Poland

## Abstract

**Introduction:**

Vitamin D deficiency is a significant problem that affects the population living in most countries. This issue is independent of place of residence, gender, age or skin color. It is mainly influenced by the climate we live in and anti-healthy lifestyle, including bad eating habits.

**Objectives:**

The aim of the study was to evaluate lipid profile, glucose level, vitamin D level, taking into account sociodemographic variables, smoking and alcohol consumption. The study also included assessment of depression taking into account sociodemographic variables and vitamin D levels in perimenopausal women.

**Methods:**

The study was conducted in a group of 191 women. The study consisted of two stages. The first part was carried out by means of a diagnostic survey with the use of questionnaire technique. The survey form consisted of two parts: a questionnaire that included questions about sociodemographic data and selected medical information, and the Beck Depression Inventory. The second stage of the study involved the collection of peripheral blood from each respondent in order to determine lipid profile, glycemia and serum vitamin D levels.

**Results:**

The age of the female respondents ranged from 45 to 65 years, mean age was 53.1±5.37 years, median 53 years. Vitamin D result was below normal in 78%. 77% had elevated total cholesterol levels. 91.6% of the respondents had HDL fraction cholesterol levels within the normal range. On the other hand, 64.4% were characterized by too high LDL fraction cholesterol and 84.8% of women showed normal triglyceride levels. Among the respondents, 91.1% had normal glycemic levels. Analysis of collected data showed a weak negative correlation between serum vitamin D concentration and concentrations of: total cholesterol (rho=-0.14; p=0.05), LDL fraction cholesterol (rho=-0.16; p=0.026) and triglycerides (rho=-0.22; p=0.002) of the examined women. Only in the case of HDL fraction cholesterol (p=0.067) there was no statistically significant correlation. There was also no statistically significant correlation between serum vitamin D levels and glycemia and severity of depression (p=0.152).

**Conclusions:**

Most of the women studied did not manifest depressive disorders. Of the various factors affecting the severity of depression, only education was associated with depression severity.

Smoking adversely affects serum vitamin D concentration in the examined women.

Cessation of menstruation influenced carbohydrate metabolism and vitamin D concentration. Blood glucose concentration increased with the age of the studied women.

There was a relationship between vitamin D concentration and values of total cholesterol, LDL fraction cholesterol and triglycerides. Therefore, considering the biological functioning of the studied women, it is important to maintain normal vitamin D levels.

**Disclosure of Interest:**

None Declared

